# Genome-wide screening of DNA methylation in bovine blastocysts with different kinetics of development

**DOI:** 10.1186/s13072-017-0171-z

**Published:** 2018-01-08

**Authors:** Jessica Ispada, Camila Bruna de Lima, Marc-André Sirard, Patrícia Kubo Fontes, Marcelo Fábio Gouveia Nogueira, Kelly Annes, Marcella Pecora Milazzotto

**Affiliations:** 1 Institute of Biomedical Sciences, Universidade de São Paulo, São Paulo, Brazil; 2 Laboratório de Biologia Celular e Molecular - Bloco A – 502-3, Center of Natural and Human Sciences, Universidade Federal do ABC, Av dos Estados, 5001, Bangu, Santo André, São Paulo Brazil; 3 Centre de Recherche en Biologie de la Reproduction, Faculté des Sciences de l’Agriculture et de l’Alimentation, Université Laval, Quebec, Canada; 4 Departament of Pharmacology, Institute of Biosciences, Universidade Estadual Paulista (UNESP), Campus Botucatu, Botucatu, São Paulo Brazil; 5 Departament of Biological Sciences, School of Sciences and Languages, Universidade Estadual Paulista (UNESP), Campus Assis, Assis, São Paulo Brazil

**Keywords:** Blastocyst, Kinetics of development, Epigenetics, DNA methylation

## Abstract

**Background:**

The timing of the first cell divisions may predict the developmental potential of an embryo, including its ability to establish pregnancy. Besides differences related to metabolism, stress, and survival, embryos with different speeds of development present distinct patterns of gene expression, mainly related to energy and lipid metabolism. As gene expression is regulated by epigenetic factors, and that includes DNA methylation patterns, in this study we compared the global DNA methylation profile of embryos with different kinetics of development in order to identify general pathways and regions that are most influenced by this phenotype. For this purpose, bovine embryos were in vitro produced using sexed semen (female), classified as fast (four or more cells) or slow (two cells) at 40 hpi and cultured until blastocyst stage, when they were analyzed.

**Results:**

Genome-wide DNA methylation analysis identified 11,584 differently methylated regions (DMRs) (7976 hypermethylated regions in fast and 3608 hypermethylated regions in slow embryos). Fast embryos presented more regions classified as hypermethylated distributed throughout the genome, as in introns, exons, promoters, and repeat elements while in slow embryos, hypermethylated regions were more present in CpG islands. DMRs were clustered by means of biological processes, and the most affected pathways were related to cell survival/differentiation and energy/lipid metabolism. Transcripts profiles from DM genes connected with these pathways were also assessed, and the most part disclosed changes in relative quantitation.

**Conclusion:**

The kinetics of the first cleavages influences the DNA methylation and expression profiles of genes related to metabolism and differentiation pathways and may affect embryo viability.

**Electronic supplementary material:**

The online version of this article (10.1186/s13072-017-0171-z) contains supplementary material, which is available to authorized users.

## Background

Epigenetic mechanisms, such as DNA methylation, are regulators of the phenotype in cells that share the same genetic profile. During DNA methylation process, the *S*-adenosylmethionine (SAM) provides a methyl group that is added to the carbon 5 of cytosines by enzymes called DNA methyltransferases (Dnmt1, 3a and 3b). Most frequently, the methyl group addition occurs in dinucleotides formed by cytosine and guanine (CpG) [1, 2]. In this context, the enzyme Dnmt1 is responsible for maintenance of methylation during cell replication, while Dnmt3a and Dnmt3b establish new methylations on DNA [3]. The DNA demethylation can occur passively or actively through the activity of 10–11 translocation (Tet 1–3) enzymes [4].

DNA demethylation and methylation are essential events during embryonic development, when the embryo undergoes a wave of demethylation during the first cleavages followed by de novo methylation starting after the eight-cells stage (bovines) [5]. In addition, the embryo needs to maintain imprinted genes and transposable elements silenced through the demethylation process. This epigenetic reprogramming is crucial to transform gametes (highly methylated) in totipotent cells (lower methylated) and, subsequently, into pluripotent cells (moderately methylated) [2].

Regulation of DNA methylation and other epigenetic mechanisms control the DNA transcription (increase in methylation is usually related to gene silencing) and, consequently, phenotype, metabolism, and response to environment [6]. The use of assisted reproductive technologies (ART), while improving the number of offsprings from zootechnical important animals, can also deregulate the wave of demethylation/remethylation and affect blastocyst development, pregnancy rates, and cause offspring abnormalities [7–9].

The kinetics of the first cleavages of in vitro produced (IVP) embryos is proposed to be a tool to select the most viable ones. A recent study comparing fast and slow IVP blastocysts (based on the speed of the first cleavages) reported transcriptional changes, mainly related to energy and lipid metabolism [10]. Moreover, embryos with different kinetics of development consume and secrete different substrates even when cultured under the same conditions [10–12].

Since blastocysts originated from fast and slow cleavages present distinct phenotypes and responses to environment, in this study, we evaluated whether these blastocysts had differences in DNA methylation status. We also identified the most affected biological processes, cellular components, and molecular functions of blastocysts generated from fast- or slow-cleavage embryos and characterized the main transcripts related to affected biological process.

## Methods

### Experimental design

Bovine embryos were produced in vitro and classified as fast or slow according to the number of cells at 40 hpi (fast—4 or more cells; slow—2 cells) as described elsewhere [10]. At day 7 of in vitro culture, blastocysts were collected and kept frozen at − 80 °C. Ten embryos per replicate (four replicates) from each group were analyzed for global DNA methylation status using EmbryoGENE platform [13]. Another set of embryos was produced to validate the results (ten embryos per replicate/three replicates) by checking DNA methylation on specific/selected regions through RT-qPCR. Finally, relative quantification of transcripts related to the most affected pathways was performed using BioMark^TM^ HD of another set of embryos (three embryos per replicate/four replicates).

### In vitro production of bovine embryos

Slaughterhouse ovaries were transported in sterile saline at 30–32 °C to the laboratory. Cumulus–oocyte complexes (COC) were then collected by follicular aspiration, washed with in vitro maturation (IVM) medium [tissue culture medium (TCM)-199—bicarbonate containing 10% fetal calf serum (FCS), 50 µg/mL gentamicin sulfate, 0.2 mM sodium pyruvate, 1 mg/mL estradiol, 10 µg/mL of follicle-stimulating hormone (FSH), and 10 µg/mL of luteinizing hormone (LH)], and thereafter, incubated in 90 µL drops of IVM medium under mineral oil at 38.5 °C for 22–24 h in 5% CO_2_ and high humidity.

TALP culture medium (Tyrode’s albumin–lactate–pyruvate) used for semen purification and in vitro fertilization (IVF) was prepared according to Parrish et al. [14]. COCs were washed and transferred to drops of IVF medium [30 COCs/70 µL of modified TALP solution, 6 mg/mL bovine serum albumin (BSA)-free fatty acids, 50 µg/mL gentamicin sulfate, 0.2 mM sodium pyruvate, 17,500 USP/mL heparin, and 0.044 µL/mL PHE (2 μM penicillamine, 1 μM hipotaurina, and 0.25 mM epinephrine)]. Sexed semen straws (female) were thawed for 30 s at 37 °C and purified by Percoll gradient. After washing, each drop was inseminated with 10 µL of the resulting pellet. IVF was held at 38.5 °C and 5% of CO_2_ in high humidity for 18 h.

After IVF, presumptive zygotes were denuded and transferred to 90 µL drops of in vitro culture (IVC) medium 1 [potassium simplex optimization medium (KSOM) [15] supplemented with 9% FCS, 0.02 µL/mL gentamicin sulfate, 8 µL/mL of nonessential amino acids, and 16 µL/mL of essential amino acids]. After 40 h, the medium was replaced by IVC medium 2 [synthetic oviduct fluid (SOF) [16, 17] supplemented with 2% essential amino acids, 1% nonessential amino acids, and 5% FCS]. IVC was carried out at 38.5 °C, 5% CO_2_, and high humidity. Embryos were classified as “fast-cleavage” (four cells or more) or “slow-cleavage” (two cells) at 40 hpi. Expanded blastocysts were collected at 168 hpi and classified as FBL (blastocysts derived from “fast-cleavage” group) and SBL (blastocysts derived from “slow-cleavage” group) and stored at − 80 °C.

### Genome-wide DNA methylation analysis: EmbryoGENE DNA methylation array

EmbryoGENE DNA methylation array was performed as previously described by Shojaei-Saadi et al. [13]. Briefly, genomic DNA (gDNA) was extracted using AllPrep DNA/RNA Mini Kit (QIAGEN, Mississauga, ON, Canada). gDNA and a spike-in control DNA were digested with *Mse*I (5′-T/TAA-3′) and ligated with specific adaptors (MseLig 21: 5′-AGT GGG ATT CCG CAT GCT AGT-3′, MseLig 12: 5′-TAA CTA GCA TGC-3′). gDNA was cleaved using FastDigest™ enzyme cocktail with enzymes that recognize DNA methylation and only cleave at unmethylated sites [*Hpa*II (5′-C/CGG-3′), *HinP1*I (5′-GC/GC-3′), and *Aci*I (5′-C/CGC-3′)]. The cleavage efficiency was determined by RT-qPCR of the spiked-in control DNA. After that, samples underwent ligation-mediated polymerase chain reaction (LM-PCR) in order to amplify the methylated fragments. LM-PCR products were purified using QIAquick PCR Purification Kit (Qiagen), and adaptors were enzymatically removed by incubation with *Mse*I. Samples were labeled by using Universal Linkage System Fluorescent gDNA labeling kit [Cy-3 or Cy-5] (Kreatech Biotechnology) and hybridized on a custom-designed array slide [13]. Slides were washed as per manufacturer’s recommendation, scanned [PowerScanner (Tecan)], and analyzed with Array-Pro Analyzer 6.3 software (MediaCybernetics).

### Validation of DNA methylome

Ten blastocysts per group were subjected to three freezing/thawing cycles followed by incubation with proteinase K (200 µg/mL) for gDNA extraction. The samples were divided into three groups of 10 µL: control—DNA fragmented only with *Mse*I enzyme; *Hpa*II—DNA fragmented with *Mse*I and *Hpa*II enzymes, and *HinP1*I—DNA fragmented with *Mse*I and *HinP1*I enzymes (FastDigest-ThermoFisher Scientific™, USA). gDNA was washed with 70% ethanol, air dried, and resuspended in 50 µL of DNAse/RNAse free water. gDNA was RT-qPCR-amplified with primers (see Additional file 1) specifically designed for regions that were previously identified as differently methylated and whose amplicon contained only one site for *Hpa*II or *HinP1*I and no site for *Mse*I.

### Gene expression analysis

For gene expression analysis, 23 genes were selected based on differences in methylation status and/or participation in affected pathways. Total RNA of three blastocysts per replicate per group (four replicates) was extracted with PicoPure^®^ RNA Isolation Kit (Applied Biosystems™, USA) as described by the manufacturer. Briefly, samples were incubated with extraction buffer at 42 °C for 30 min, followed by incubation with DNAse (Qiagen, USA) at room temperature for 15 min and RNA purification with column. RNA was eluted and stored at − 20 °C for 1 day. cDNA synthesis was performed with high-capacity cDNA reverse transcription (Applied Biosystems, USA # 4368814) following manufacturer’s recommendation. Initially, to 50 ng of total RNA were added 10× RT buffer, dNTPs (25×), random primers (10× RT), reverse transcriptase (MultiScribe^®^), and DNAse/RNAse free water. Incubation was held at 25 °C for 10 min, 37 °C for 120 min, and 85 °C for 5 min.

cDNA was preamplified in a mix containing all TaqMan assays (see Additional file 2) (Applied Biosystems, USA). Chip preparation was performed as described by manufacturer (Fluidigm, USA) as follows: Solution containing TaqMan assays and Assay Loading Reagent (Fluidigm, USA) was allocated at the assay region. Solution containing TaqMan Master Mix (Applied Biosystems, USA), Sample Loading Reagent (Fluidigm, USA), and preamplified cDNA was allocated at the sample region. The chip was transferred to BioMark™ HD Real-Time PCR (Fluidigm, USA) for RT-qPCR according to the TaqMan GE 96 × 96 Standard protocol.

### Statistical analysis

Cleavage and blastocyst data were assessed for normality distribution by D’Agostino–Pearson test and subsequently analyzed by two-tailed Student’s *t* test with *α* = 5%.

Methylome data analysis was performed as described before by Shojaei-Saadi et al. [13]. Briefly, probes that showed signal intensity above the background based on the intensity cutoff (mean + 4 * SD) of the negative controls were normalized, fitted to a linear model, and differences in DNA methylation were inferred by Bayesian statistics using Limma Bioconductor package version 3.6.9. Differently methylated regions (DMRs) were considered as fragments of DNA with multiple CpG sites that presented *P* < 0.05 and log2 (fold-change) ≥ 1.5 in methylation between groups, even if the signal above background was not observed in all four replicates and regardless of the amount of CpGs present in the fragment. It is important to highlight that more than 95% of all DMRs presented three or four samples above background. Comparisons between FBL and SBL were performed by Bioconductor MADE4 package. Venn diagram and volcano plot show the amount of probes above the background and DMRs between groups, respectively. Circular plot was created with arbitrary fold-change and *P* value thresholds at the ~ 15,000 most significant elements identified in EmbryoGENE DNA methylation array, showing the most variable elements.

Different enrichments analysis was performed through a series of integrated scripts that categorize the information based on: CpG island density, CpG island length, CpG island distance, genomic location, and repetitive element types, aiming at the observation of DNA methylation on a genomic scale. These scripts belong to the friendly version provided by EmbryoGENE methylation analysis pipeline (EMAP) and were performed using R version 2.12.1 and Limma package using the raw data. The complete EMAP information can be reached at the EmbryoGENE site (http://emb-bioinfo.fsaa.ulaval.ca/bioinfo/html/epigenetics/Epigenetics%20Analysis%20Pipeline.pdf). Regions with higher signal intensity in the FBL compared to the SBL group were inferred as hypermethylated in FBL and hypomethylated in SBL, while regions with higher signal intensity in SBL compared to the FBL were inferred as hypermethylated in SBL and hypomethylated in FBL. These regions were designated as hypermethylated or hypomethylated regions for brevity.

Genes were classified as differently methylated when one associated probe, localized in the gene body (promoter, exon or intron), was identified as a differently methylated probe. If more than one probe was associated with the same gene, the one with highest fold-change and lowest *P* value was selected. David functional annotation analysis was also performed for each list of genes (2325 hypermethylated and 1207 hypomethylated genes) obtained from the comparison FBL × SBL. The DMRs were classified and evaluated according to three different categories: biological processes, cellular components, and molecular functions.

Validation of EmbryoGENE DNA methylation array was made using the difference between the *C*_t_ values of undigested gDNA and the *C*_t_ values of the digested ones (Δ*C*_t_ = *C*_t_ digested − *C*_t_ undigested) of seven DMRs. The DMRs were selected based on its gene location, presence or absence of CpG island, and status of methylation, focusing in diversifying all these parameters to improve the reliability of the microarray data. The percentage of DNA methylation was inversely proportional to the value obtained for enzymatic digestion with *Hpa*II or *HinP1*I.

For transcripts analysis, *C*_t_ value for each target was normalized by three endogenous controls (GAPDH, ACTB, and PPIA) selected with NormFinder [18]. Δ*C*_t_ values were calculated, tested for outlier detection, and submitted to Student’s *t* test by Prism 5 software (GraphPad Software Inc., USA). For transcripts analysis, *P* values were considered as significant when *P* ≤ 0.05 [19].

## Results

### In vitro production of embryos

In vitro production rates were determined for fast and slow embryos based on the proportion of total embryos that cleave (58.0 ± 3.4) and become blastocyst (16.0 ± 2.8). There was no difference in the cleavage rate (FBL: 45.1 ± 3.0 and SBL: 54.9 ± 3.0; *P* = 0.41) or percentage of cleaved embryos that reached blastocyst stage (FBL: 55.1 ± 4.6 and SBL: 44.9 ± 4.6; *P* = 0.26) between fast- and slow-cleavage groups (Table 1).

**Table 1:** Cleavage rate and percentage of cleaved embryos that reached the blastocyst stage in FBL and SBL

	% (No.) cleavage			% (No.) blastocyst		
IVP	58.0 ± 3.4 (1362/2480)			16.0 ± 2.8 (233/1362)		
	FBL	45.1 ± 3.0 (611/1362)	*P* value 0.41	FBL	55.1 ± 4.6 (129/233)	*P* value 0.26
	SBL	54.9 ± 3.0 (751/1362)		SBL	44.9 ± 4.6 (104/233)	

### Genome-wide DNA methylation profile

**Fig. 1:**
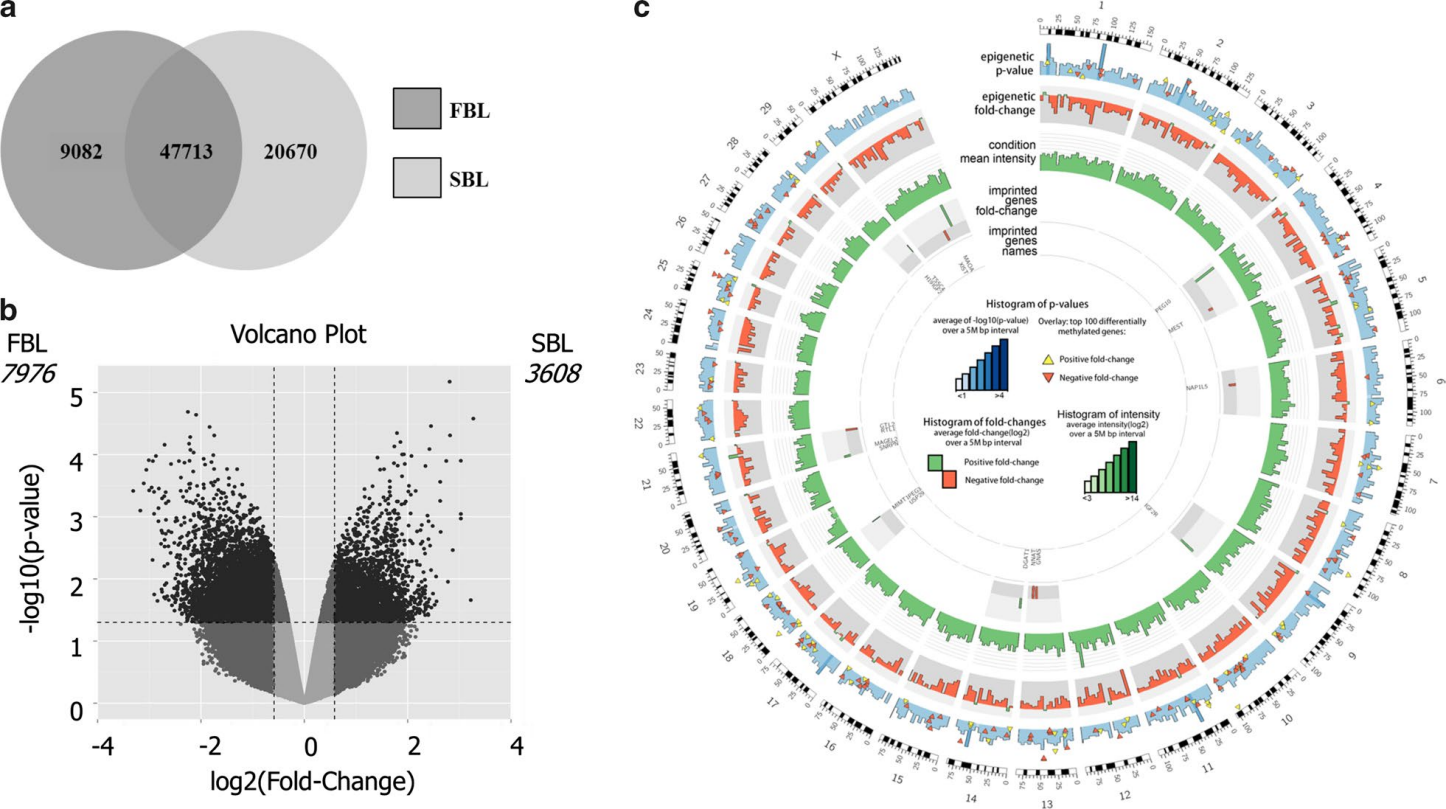
**a** Venn diagram representing probes expressed above background for FBL and SBL. **b** Volcano plot showing DMRs hypermethylated in FBL (left—7976) and hypermethylated in SBL (right—3608). Each dot represents a DMR, being positioned according to its values of log2 (fold-change) and *P* value. **c** Circular plot showing the most significant DMRs distributed among chromosomes and imprinted regions. *P* < 0.05 and log2 (fold-change) ≥ 1.5

DNA methylome array was used to identify differentially methylated regions between fast- and slow-developing groups. From 414,566 probes, 9082 were above the background only in FBL, 20,670 only in SBL and 47,713 in both groups (Fig. 1a). FBL presented a higher number of hypermethylated regions than SBL (7976 vs. 3608) (Fig. 1b). DMRs were distributed in all chromosomes, and the analysis of imprinted genes showed that these genes were hypermethylated (six paternally and two maternally imprinted) and hypomethylated (four paternally and two maternally imprinted) in FBL in comparison with SBL (Fig. 1c).

**Fig. 2:**
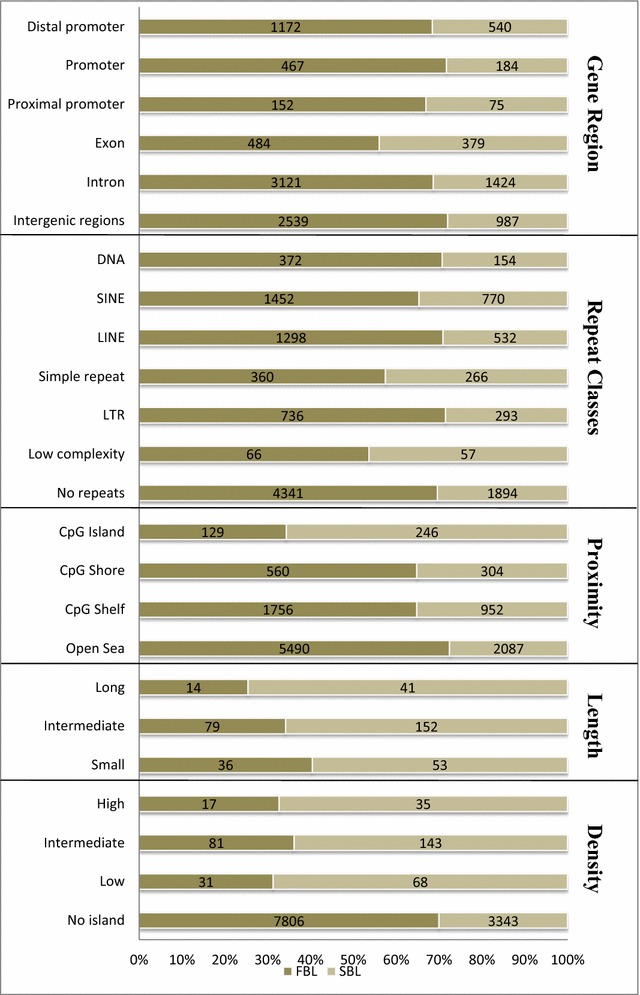
Localization of DMRs hypermethylated based on CpGs islands distance, length, density, genic regions, and repetitive elements in FBL and in SBL. *P* < 0.05 and log2 (fold-change) ≥ 1.5

In FBL group, more regions classified as hypermethylated were present in distal promoters, promoters, proximal promoters, exonic, intronic, and intergenic regions than in SBL (Fig. 2). Also, a higher number of hypermethylated regions were present in FBL in repetitive elements, such as short-interspersed repetitive elements (SINEs), long-interspersed repetitive elements (LINEs), simple repeats, long-terminal-repeat (LTR) retrotransposons, and low-complexity repetitive elements than in SBL (Fig. 2). More hypermethylated regions in FBL were also located in CpG shore, CpG shelf, and open sea than SBL. On the other hand, SBL exhibited more hypermethylated regions in CpGs island than FBL regardless of CpGs islands density of CGs (high, intermediate, and low density) or length (long, intermediate, and small) (Fig. 2). DMRs were distributed to the whole gene body in both groups, but it is important to highlight that the major amount of DMRs were located in introns (total of 4545 DMRs) and distal promoter (total of 1722 DMRs) (Fig. 2).

### Microarray validation

EmbryoGENE DNA methylation array validation was performed by DNA methylation-sensitive restriction digestion followed by RT-qPCR for seven DMRs to verify if the array platform worked properly. Six out of seven amplified regions presented the same pattern of DNA methylation observed when using EmbryoGENE between FBL and SBL (Table 2).

**Table 2:** Percentage of DNA methylation of DMRs chosen to validate the microarray

DMRs	Percentage of DNA methylation in FBL	Percentage of DNA methylation in SLB	Microarray results
edma_met_11_10337	39.1	55.4	Hypermethylated in SBL
edma_met_03_16369	12.3	50.6	Hypermethylated in SBL
edma_met_20_08418	19.1	100	Hypermethylated in SBL
edma_met_13_05328	100	38.0	Hypermethylated in SBL
edma_met_02_07813	100	74.7	Hypermethylated in FBL
edma_met_10_01759	99.5	59.8	Hypermethylated in FBL
edma_met_21_11309	78.8	48.9	Hypermethylated in FBL

### Functional annotation

Functional annotation of differentially methylated gene revealed the biological functions, cellular components, and molecular functions most affected by morphokinetics. Regarding biological processes, 101 pathways were identified as hypermethylated in FBL, as well as 44 cellular components and 49 molecular functions in FBL in comparison with SBL. In SBL, 27 pathways of biological processes, 8 cellular components, and 27 molecular functions were identified as hypermethylated in SBL in comparison with FBL. The most relevant annotations identified as hypermethylated are represented in Table 3 (see Additional file 3 containing list of genes) for FBL and Table 4 (see Additional file 4 containing list of genes) for SBL.

**Table 3:** Hypermethylated annotations in fast-cleavage embryos

Term	Number of genes hypermethylated	*P* value
*Biological process*		
Phosphate metabolic process	134	2.21E−09
Intracellular signaling cascade	83	6.16E−03
Ionic transport	80	3.08E−02
Macromolecule catabolic process	55	2.22E−02
Regulation of programmed cell death	51	2.06E−02
Regulation of cell proliferation	47	2.69E−02
Regulation of small GTPase-mediated signal transduction	43	3.40E−06
Cytoskeleton organization	29	7.01E−02*
Cellular component morphogenesis	25	2.48E−02
Fatty acid metabolic process	21	7.84E−03
*Cellular components*		
Plasma membrane	171	2.30E−03
Organelle lumen	88	9.56E−03
Endoplasmic reticulum	65	4.48E−02
Golgi apparatus	61	6.39E−03
Cytosol	50	2.61E−03
*Molecular function*		
Ion biding	388	7.47E−06
Nucleotide biding	273	3.13E−06
ATP biding	199	2.58E−09
Protein kinase activity	102	9.08E−10
RNA biding	57	5.78E−02

* Represent tendency (*P* value > 0.05 and ≤ 0.1)

**Table 4:** Hypermethylated annotations in slow-cleavage embryos

Term	Number of genes hypermethylated	P value
*Biological process*		
Phosphorus metabolic process	55	2.66E−02
RNA processing	27	2.10E−02
Regulation of small GTPase-mediated signal transduction	24	2.89E−04
Generation of precursor metabolites and energy	20	8.53E−02*
Regulation of Ras protein signal transduction	18	2.82E−03
Chordate embryonic development	17	5.94E−02*
Cellular component morphogenesis	15	3.13E−02
Secretion	14	4.17E−02
Cellular proliferation	13	5.25E−02
Blastocyst development	8	5.14E−03
*Cellular component*		
Plasma membrane	88	1.57E−02
Mitochondrion	58	1.74E−02
Endoplasmatic reticulum	34	9.59E−02*
Golgi apparatus	14	4.16 E−02
Spliceosome	10	2.77 E−02
*Molecular function*		
Nucleotide biding	135	9.46E−04
ATP biding	84	1.53E−02
Protein kinase activity	44	2.11E−03
GTPase regulator activity	30	8.52E−05
FAD biding	9	1.79E−02

* Represent tendency (*P* value > 0.05 and ≤ 0.1)

### Parallel analysis of DNA methylation and transcriptional status

Based on biological process identified as differently methylated, we selected 23 genes for transcription profile analysis and also to correlate with DNA methylation status. These genes were selected from biological processes identified in the functional annotation analysis as containing differentially methylated genes considering their importance for embryo development and viability. Ten genes related to stress/cell death (BAX, BID, CASP9, DDIT3, FOXO3, GPX1, HSPA1A, NFE2L2, NOS2, and TXNRD1); four genes involved in the control of cell differentiation (NANOG, POU5F1, SALL4, and SOX2); and nine genes related to lipid metabolism (ACSL3, ACSL6, ELOVL6, FADS2, FASN, PPARα, PPARγ, PTGS2, and SCD) were selected for this purpose. We also tried to pick up hyper- and hypomethylated genes from both groups, as well as genes which did not presented differences in methylation status as we were interested in analyzing the correlation of gene expression from a specific pathway with the methylation status, whatever it was.

**Fig. 3:**
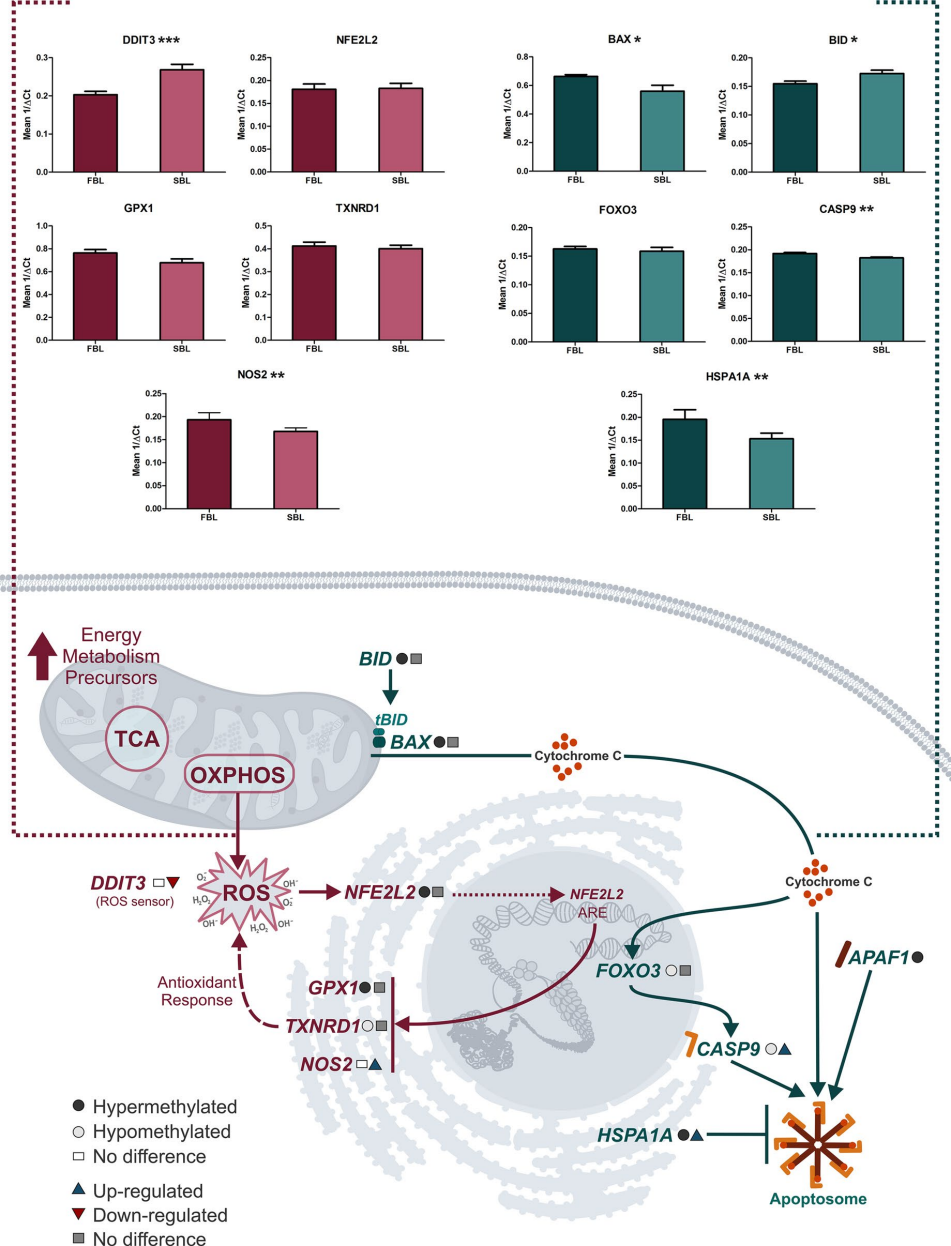
Hypothetical pathway related with cell stress and death identified with altered DNA methylation and/or genes transcripts in the FBL in comparison with SBL. Black circle represents hypermethylated genes; gray circle represents hypomethylated genes; white circle represents genes without difference in DNA methylation; blue triangle represents genes up-regulated; red triangle represents genes down-regulated; gray square represents genes without difference in RNA levels. Graphs show the difference in expression of DDIT3, NFE2L2, GPX1, TXNRD1, NOS2, BAX, BID, FOXO3, CASP9, and HSPA1A for FBL and SBL (*P ≤ 0.1; **P ≤ 0.05; ***P ≤ 0.01)

**Fig. 4:**
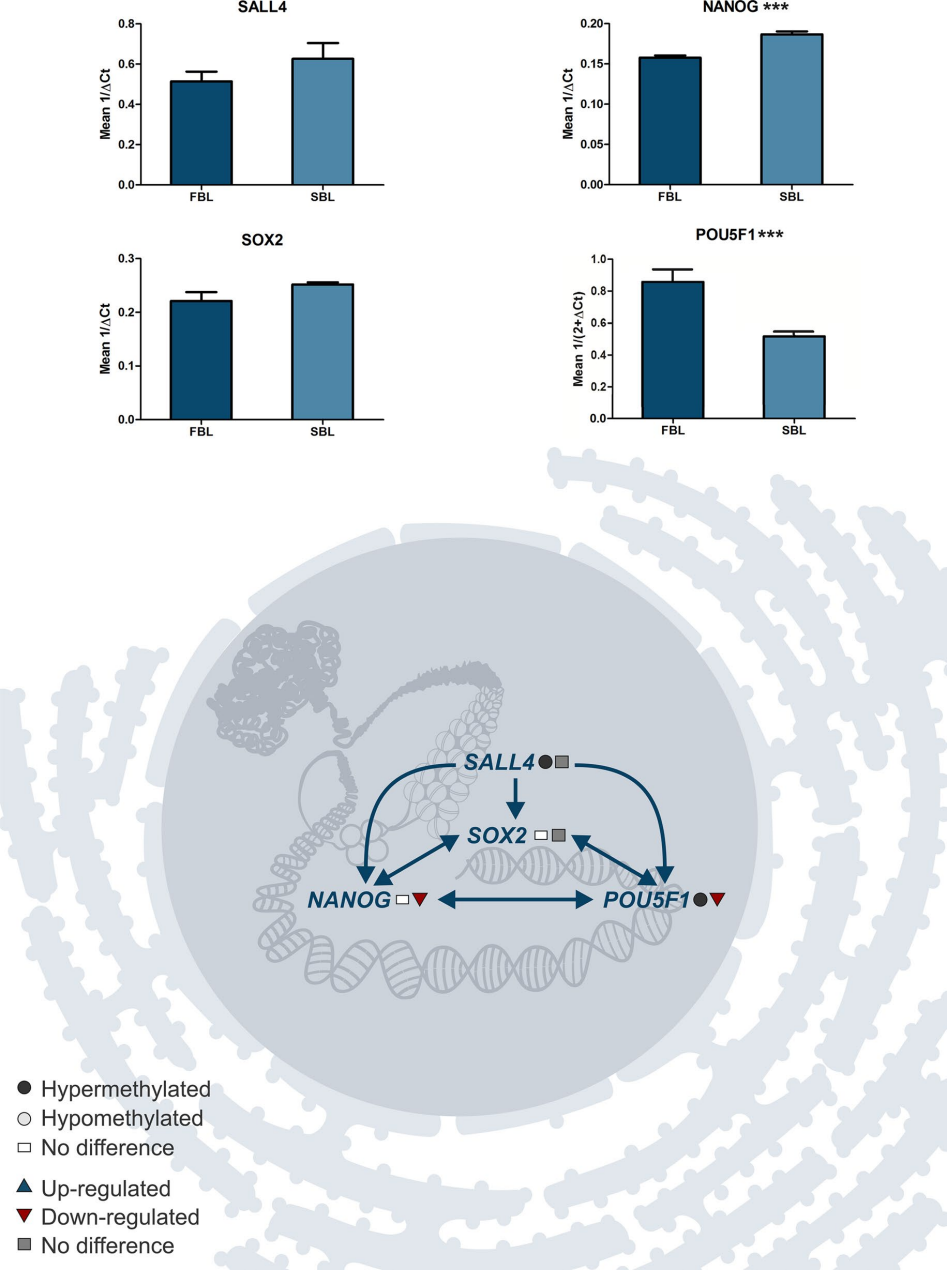
Pluripotency pathway identified with altered DNA methylation and/or genes transcripts in the FBL in comparison with SBL. Black circle represents hypermethylated genes; gray circle represents hypomethylated genes; white circle represents genes without difference in DNA methylation; blue triangle represents genes up-regulated; red triangle represents genes down-regulated; gray square represents genes without difference in RNA levels. Graphs show the difference in expression of SALL4, NANOG, SOX2, and POU5F1 for FBL and SBL (*P ≤ 0.1; **P ≤ 0.05; ***P ≤ 0.01)

**Fig. 5:**
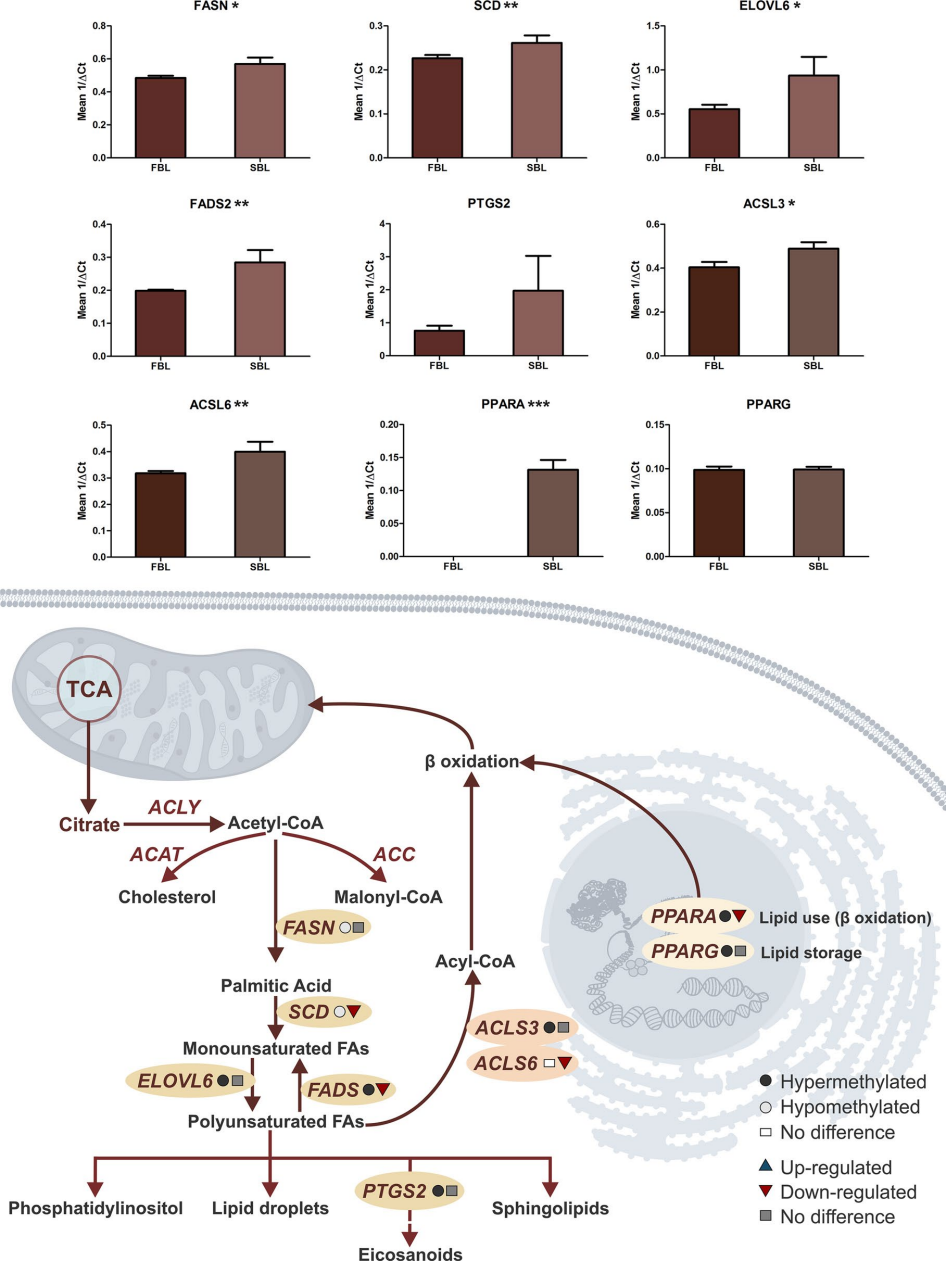
Proposed pathway of lipid metabolism identified with altered DNA methylation and/or genes transcripts in the FBL and, consequently, hypomethylated in SBL. Black circle represents hypermethylated genes; gray circle represents hypomethylated genes; white circle represents genes without difference in DNA methylation; blue triangle represents genes up-regulated; red triangle represents genes down-regulated; gray square represents genes without difference in RNA levels. Graphs show the difference in expression of FASN, SCD, ELOVL6, FADS2, PTGS2, ACSL3, ACSL6, PPARα, and PPARγ for FBL and SBL (*P ≤ 0.1; **P ≤ 0.05; ***P ≤ 0.01)

Regarding the gene expression results, five of the selected genes were hypermethylated in SBL, and from these one was overexpressed (SCN) and four did not differ statistically (TXNRD1, FOXO3, FASN, and PTGS2). Another thirteen genes were hypermethylated in FBL, and from these, two were overexpressed (POU5F1 and HSPA1A), two were underexpressed (FADS, and PPARα), and nine did not present statistical differences (BAX, BID, ELOVL6, ACSL3, GPX1, NFE2L2, APAF1, SALL4, and PPARγ. Five genes did not present difference in methylation status, and from these, three were overexpressed in SBL (DDIT3, NANOG, and ACSL6), NOS2 was overexpressed in FBL, and SOX2 did not differ statistically (Figs. 3, 4, 5).

## Discussion

Selection of embryos with higher potential to generate pregnancy is important to improve the success of IVP, and in this context, morphokinetics has been used as a promising tool. Recent studies showed that embryos with distinct kinetics of development present differences related to DNA damage [20], consumption of subtracts [10], secretion of metabolites [11], energy storage [10], activity of antioxidant enzymes, [21] cell survival [22], and transcription profile [10].

Although limited data regarding the epigenetic profile of these embryos is available, the elucidation of mechanisms responsible for the maintenance of these phenotypes can help the understanding of the preimplantation embryo development. Besides, it has already been well established that assisted reproduction techniques, such as superovulation protocols, in vitro production of embryos, and cryopreservation, may affect the epigenetic pattern in gametes, embryos, and future offspring [8]. Thus, in the present study, we evaluated how the kinetics of the first cleavages may influence total DNA methylation profile in bovine embryos produced in vitro, aiming to identify general pathways and regions most influenced by our phenotype. As male and female embryos exhibit distinct epigenetic reprogramming patterns [5], in the present study we excluded sex interference by using sexed semen for IVF, in order to determine DNA methylation differences exclusively related to developmental kinetics.

It is known that during early embryonic development, with exception of some imprinted genes and repetitive elements, the male and the female genome undergoes a wave of DNA demethylation during the first cleavages [23]. These events are responsible for the generation of totipotent blastomeres (highly demethylated) from specialized cells (gametes-highly methylated) [24]. According to our results, total DNA methylation was higher in FBL than in SBL. Several causes may explain this difference, such as the inability to promote correct demethylation during early cleavages or the early/late activation of de novo methylation promoted by Dnmts enzymes family. Any changes in these events might lead to failure in the methylation wave, compromising the viability of the blastomeres [2, 25].

Previous studies have already demonstrated that there are differences in DNA methylation profile between in vitro and in vivo produced embryos [26]. Although the methylation pattern of CpG islands is similar, in vitro produced embryos exhibit higher DMRs in promoters, exons, introns, and repetitive elements such as LINE, SINE, LTR, and low-complexity repeats [26]. In the present study, FBL group presented more hypermethylation in all gene regions, including repetitive elements, while SBL was more hypermethylated in CpG islands. The results suggest that fast-cleavage embryos seem to be more susceptible to a nonoptimized in vitro culture system, leading to higher levels of DNA methylation in genomic regions and compromising the gaining of de novo methylation at CpG islands, characteristics that have already been described as being generated in more stressful conditions [26].

Genomic enrichment analysis pointed out to several crucial pathways for the embryo development that were differentially methylated between groups. In FBL, hypermethylated pathways included cell death, differentiation, and lipid metabolism. Although the role of DNA methylation on gene silencing is dependent on the region where it is abundant [2], we decided to further investigate the correlation between methylation status and transcription of specific genes involved in the cited metabolic pathways. Thus, some of these genes were assessed by RT-qPCR to verify their relative expression.

As previously mentioned, cell death was one of the pathways found hypermethylated in FBL when compared to SBL. It is known that blastocysts activate apoptotic mechanisms usually through the mitochondrial activation pathway when exposed to stressful conditions [27]. This mechanism, while potentially harmful since it can drastically reduce blastomeres number, is also a way to prevent a damaged cell from generating affected daughter cells [28]. In this work, besides differences in methylation status, the initiator caspase CASP9 was up-regulated in FBL. Despite that, BID, which is responsible for cytochrome c releasing from mitochondria into cytosol [29], and APAF1, which also composes the apoptosomic complex with caspase 9 and cytochrome c, [30] were hypermethylated in FBL. Moreover, HSPA1A, which inhibits translocation of BAX, cytochrome c release, and apoptosome activity, was also up-regulated [31] (Fig. 3). Together, these results suggest that although the proapoptotic stimuli are present in FBL, the transcripts of effector proteins seem to regulate the progression of cell death. Corroborating these results, previous study of our group did not found changes in DNA fragmentation and caspase 3 and 7 activity in FBL and SBL [22].

Pathways related to cellular differentiation were also hypermethylated in FBL. The pluripotency is mostly maintained through the control of POU5F1 (OCT4), SALL4, SOX2, and NANOG [32]. In mouse, the formation of inner cell mass (ICM) is also regulated by these four genes [33]. Also, of the two genes (NANOG and SOX2) that did not differ in gene methylation between groups, only NANOG was underexpressed in FBL, while SOX2 did not differ (Fig. 4). That could suggest that FBL is further differentiated from SBL, since they differ in levels of genes transcripts that maintain pluripotency [32] and higher levels of DNA methylation, another characteristic of advanced differentiation in cells [2].

Regarding lipid metabolism, we highlight the strong association between the methylation status and the gene expression results. Lipid metabolism in preimplantation embryo is important for energy storage, blastomeres structure, membranes function [34], and survival rates after cryopreservation [35]. Differences in lipid content have been previously described in fast and slow embryos [10]. In fact, although the number of lipid droplets in FBL is higher [10], the lipid metabolism seems to be more active in SBL [12]. The results presented here corroborate with these data and suggest that the most active lipid pathway in SBL do not involve the cholesterol metabolism but rather the synthesis of fatty acids, since key enzymes are hypomethylated and/or overexpressed in SBL as ACL3, ELOV6, and FADS2 (Fig. 5). The hypomethylation and overexpression of PPARα also suggest an intense lipid metabolism, since it is expressed predominantly in tissues with high rates of fatty acid catabolism [36].

It is noteworthy that DNA methylation analysis through EmbryoGENE provides the intensity of fluorescence differentially released by DNA fragments based on relative amounts of DNA methylation. Therefore, when a DMR is identified as hypermethylated, it is possible affirm that the region has more methylations; however, we cannot determine whether these methylations will repress the gene. In part, that might explain the absence of correlation between methylation and transcription pattern for some genes. This might also be explained due to the presence of microRNAs and other posttranslational modifications such as histone modifications [2, 8]. Among the results obtained for DNA methylation in this work, some DMRs were classified as belonging to microRNAs and some histone regulators, corroborating the hypothesis that other epigenetic mechanisms may also be acting.

## Conclusions

Fast- and slow-cleavage embryos present differences in global DNA methylation profile. These differences are mainly related to distinct metabolic activity, cell structure, survival, and death. Together, this information suggests that blastocysts generated by embryos with different kinetics of development are activating and deactivating mechanisms that might lead to successful or failure in generating pregnancies. Further investigation remains necessary, mainly to understand the role of other epigenetic mechanisms and the DNA methylation establishment through embryonic development in fast- and slow-cleavage embryos. Hopefully, this information will demonstrate whether these embryos are activating or deactivating mechanisms to regulate DNA methylation. Also, it is essential to clarify whether these epigenomic alterations are cause or consequence of differences in phenotype and/or response to environment and how these may aid the identification of embryos with better quality and potential to generate pregnancies.

## Additional files

**Additional file 1.** Primers designed to validate DMRs and detailed results obtained for each primer.

**Additional file 2.** Transcripts analyzed to determine the correlation between DNA methylation and RNA expression.

**Additional file 3.** Hypermethylations in FBL.

**Additional file 4.** Hypermethylations in SBL

## Abbreviations

UNESP: Universidade Estadual Paulista
IVP: in vitro production
DMRs: differently methylated regions
SAM: *S*-adenosylmethionine
ART: assisted reproductive technologies
COC: cumulus–oocyte complexes
IVM: in vitro maturation
TCM: tissue culture medium
FCS: fetal calf serum
FSH: stimulating hormone
LH: luteinizing hormone
TALP: tyrode’s albumin–lactate–pyruvate
IVF: in vitro fertilization
BSA: bovine serum albumin
PHE: penicillamine hipotaurina epinephrine
IVC: in vitro culture
KSOM: potassium simplex optimization medium
SOF: synthetic oviduct fluid
gDNA: genomic DNA
LM-PCR: ligation-mediated polymerase chain reaction
RT-qPCR: real-time quantitative polymerase chain reaction
SINE: short-interspersed repetitive elements
LINE: long-interspersed repetitive elements
LTR: long-terminal-repeat
FBL: fast blastocyst
SBL: slow blastocyst
ICM: inner cell mass
