# Supplementary material for: Genome-wide screening of DNA methylation in bovine blastocysts with different kinetics of development
Source: Epigenetics Chromatin. 2018 Jan 8;11:1. doi: 10.1186/s13072-017-0171-z (PMC5757301; doi:10.1186/s13072-017-0171-z)
Supplement: Supplementary file 1 — Additional file 1. Primers designed to validate DMRs and detailed results obtained for each primer. [file 13072_2017_171_MOESM1_ESM.docx]

Primers designed to Validate EmbryoGENE DNA methylation array.

| **DMR** | **Primer Forward** | **Primer Reverse** | **Amplicon Length** |
| --- | --- | --- | --- |
| edma_met_11_10337 | gcacgactcccttctagaca | ggcagaggatctacagcca | 84 |
| edma_met_03_16369 | cacatgtagaactggccca | ttctcctctacgaccctaggta | 91 |
| edma_met_20_08418 | ttctcctctacgaccctaggta | gaagttgccccgttacttct | 97 |
| edma_met_13_05328 | gaagttgccccgttacttct | ctgacgttccaggagacc | 83 |
| edma_met_02_07813 | ccataatgacacacactccgtt | aggctctatgggacagca | 87 |
| edma_met_10_01759 | aggctctatgggacagca | gtcttctggaagcacctcc | 84 |
| edma_met_21_11309 | gtcttctggaagcacctcc | ccagaggagctgtagatcct | 113 |

Complete results for edma_met_11_10337 (P= 0,10)

| **Group** | **Sample** | **Ct** | **ΔCt** | **ΔCt Mean** | **Percentage of digestion** | **Percentage of methylation** |
| --- | --- | --- | --- | --- | --- | --- |
| FBL 1 | Control | 28.86 | 0.93 | 1.350 ± 0.325 | 60.9 | **39.1** |
|  | Digested | 29.79 |  |  |  |  |
| FBL 2 | Control | 30.82 | 1.13 |  |  |  |
|  | Digested | 31.95 |  |  |  |  |
| FBL 3 | Control | 29.75 | 1.99 |  |  |  |
|  | Digested | 31.74 |  |  |  |  |
| SBL 1 | Control | 28.31 | 0.84 | 0.850 ± 0.061 | 44.6 | **55.4** |
|  | Digested | 29.15 |  |  |  |  |
| SBL 2 | Control | 31.69 | 0.96 |  |  |  |
|  | Digested | 32.65 |  |  |  |  |
| SBL 3 | Control | 31 | 0.75 |  |  |  |
|  | Digested | 31.75 |  |  |  |  |

Complete results for edma_met_03_16369 (P= 0,02)

| Group | Sample | Ct | ΔCt | ΔCt Mean | Percentage of digestion | Percentage of methylation |
| --- | --- | --- | --- | --- | --- | --- |
| FBL 1 | Control | 26.94 | 3.62 | 2.987 ± 0.684 | 87.7 | **12.3** |
|  | Digested | 28.56 |  |  |  |  |
| FBL 2 | Control | 27.8 | 3.72 |  |  |  |
|  | Digested | 31.52 |  |  |  |  |
| FBL 3 | Control | 27.24 | 1.62 |  |  |  |
|  | Digested | 30.86 |  |  |  |  |
| SBL 1 | Control | 26.06 | 0.97 | 0.983 ± 0.139 | 49.4 | **50.6** |
|  | Digested | 27.03 |  |  |  |  |
| SBL 2 | Control | 26.02 | 1.23 |  |  |  |
|  | Digested | 27.25 |  |  |  |  |
| SBL 3 | Control | 31.03 | 0.75 |  |  |  |
|  | Digested | 31.78 |  |  |  |  |

Complete results for edma_met_20_08418 (P= 0,02)

| Group | Sample | Ct | ΔCt | ΔCt Mean | Percentage of digestion | Percentage of methylation |
| --- | --- | --- | --- | --- | --- | --- |
| FBL 1 | Control | 26.47 | 3.91 | 2.377 ± 1.010 | 80.9 | **19.1** |
|  | Digested | 30.38 |  |  |  |  |
| FBL 2 | Control | 26.69 | 0.47 |  |  |  |
|  | Digested | 27.16 |  |  |  |  |
| FBL 3 | Control | 32.62 | 2.75 |  |  |  |
|  | Digested | 35.37 |  |  |  |  |
| SBL 1 | Control | 29.28 | \| -1.17 \| \| --- \| | -0.887 ± 0.382 | 0 | **100** |
|  | Digested | 28.11 |  |  |  |  |
| SBL 2 | Control | 29.43 | \| -0.13 \| \| --- \| |  |  |  |
|  | Digested | 29.3 |  |  |  |  |
| SBL 3 | Control | 31.76 | -1.36 |  |  |  |
|  | Digested | 30.4 |  |  |  |  |

Complete results for _met_13_05328 (P= 0,04)

| Group | Sample | Ct | ΔCt | ΔCt Mean | Percentage of digestion | Percentage of methylation |
| --- | --- | --- | --- | --- | --- | --- |
| FBL 1 | Control | 29.86 | -1.00 | -0.300 ± 0.665 | 0 | **100** |
|  | Digested | 28.86 |  |  |  |  |
| FBL 2 | Control | 30.73 | 1.03 |  |  |  |
|  | Digested | 31.76 |  |  |  |  |
| FBL 3 | Control | 31.87 | -0.93 |  |  |  |
|  | Digested | 30.94 |  |  |  |  |
| SBL 1 | Control | 27.19 | 1.59 | 1.387 ± 0.218 | 62.0 | **38.0** |
|  | Digested | 28.78 |  |  |  |  |
| SBL 2 | Control | 27.13 | 1.62 |  |  |  |
|  | Digested | 28.75 |  |  |  |  |
| SBL 3 | Control | 31.9 | 0.95 |  |  |  |
|  | Digested | 32.85 |  |  |  |  |

Complete results for edma_met_02_07813 (P= 0,07)

| Group | Sample | Ct | ΔCt | ΔCt Mean | Percentage of digestion | Percentage of methylation |
| --- | --- | --- | --- | --- | --- | --- |
| FBL 1 | Control | 29.86 | 0.34 | -0.420 ± 0.448 | 0 | **100** |
|  | Digested | 30.2 |  |  |  |  |
| FBL 2 | Control | 31.65 | -0.39 |  |  |  |
|  | Digested | 31.26 |  |  |  |  |
| FBL 3 | Control | 33.28 | -1.21 |  |  |  |
|  | Digested | 32.07 |  |  |  |  |
| SBL 1 | Control | 31.14 | 0.25 | 0.423 ± 0.118 | 25.3 | **74.7** |
|  | Digested | 31.39 |  |  |  |  |
| SBL 2 | Control | 32.19 | 0.37 |  |  |  |
|  | Digested | 32.56 |  |  |  |  |
| SBL 3 | Control | 29.74 | 0.65 |  |  |  |
|  | Digested | 30.39 |  |  |  |  |

Complete results for edma_met_10_01759 (P= 0,09)

| Group | Sample | Ct | ΔCt | ΔCt Mean | Percentage of digestion | Percentage of methylation |
| --- | --- | --- | --- | --- | --- | --- |
| FBL 1 | Control | 33.02 | 0.49 | 0.009 ± 0.446 | 0.5 | **99.5** |
|  | Digested | 29.53 |  |  |  |  |
| FBL 2 | Control | 29.68 | 0.4 |  |  |  |
|  | Digested | 26 |  |  |  |  |
| FBL 3 | Control | 28.72 | -0.89 |  |  |  |
|  | Digested | 26.07 |  |  |  |  |
| SBL 1 | Control | 26.59 | 0.97 | 0.743 ± 0.115 | 40.2 | **59.8** |
|  | Digested | 27.56 |  |  |  |  |
| SBL 2 | Control | 32.74 | 0.66 |  |  |  |
|  | Digested | 33.4 |  |  |  |  |
| SBL 3 | Control | 29.26 | 0.60 |  |  |  |
|  | Digested | 29.86 |  |  |  |  |

Complete results for edma_met_21_11309 (0,03)

| Group | Sample | Ct | ΔCt | ΔCt Mean | Percentage of digestion | Percentage of methylation |
| --- | --- | --- | --- | --- | --- | --- |
| FBL 1 | Control | 28.08 | -0.03 | 0.343 ± 0.259 | 21.2 | **78.8** |
|  | Digested | 28.05 |  |  |  |  |
| FBL 2 | Control | 27.47 | 0.84 |  |  |  |
|  | Digested | 28.31 |  |  |  |  |
| FBL 3 | Control | 31.06 | 0.84 |  |  |  |
|  | Digested | 31.28 |  |  |  |  |
| SBL 1 | Control | 26.53 | 1.03 | 1.030 ± 0.029 | 51.1 | **48.9** |
|  | Digested | 27.56 |  |  |  |  |
| SBL 2 | Control | 26.49 | 1.08 |  |  |  |
|  | Digested | 27.57 |  |  |  |  |
| SBL 3 | Control | 29.57 | 0.98 |  |  |  |
|  | Digested | 30.55 |  |  |  |  |
