# Supplementary material for: Genome-wide screening of DNA methylation in bovine blastocysts with different kinetics of development
Source: Epigenetics Chromatin. 2018 Jan 8;11:1. doi: 10.1186/s13072-017-0171-z (PMC5757301; doi:10.1186/s13072-017-0171-z)
Supplement: Supplementary file 2 — Additional file 2. Transcripts analyzed to determine the correlation between DNA methylation and RNA expression. [file 13072_2017_171_MOESM2_ESM.docx]

| **Gene** | **ThermoFisher TaqMan code** | **Gene ID UniGene** | **Gene** |
| --- | --- | --- | --- |
| ACSL3 | Acyl-CoA Synthetase 3 | Bt04282138_m1 | Bt.89521 |
| ACSL6 | Acyl-CoA Synthetase 6 | Bt03231692_m1 | Bt.11904 |
| ACTB | actin, beta | PA5-16914 | Bt.14186 |
| BAX | BCL2-Associated X Protein | Bt03211777_g1 | Bt.109788 |
| BID | BH3 Interacting Domain Death Agonist | Bt03241255_m1 | Bt.87470 |
| CASP9 | Caspase 9, apoptosis-related cysteine peptidase | Bt04282453_m1 | Bt.66332 |
| DDIT3 | DNA-damage-inducible transcript 3 | Bt03251320_g1 | Bt.65257 |
| ELOVL6 | Fatty acid elongase 6 | Bt00907566_m1 | Bt.2073 |
| FADS | Fatty acid desaturase 2 | Bt03256255_g1 | Bt.3891 |
| FASN | Fatty acid synthase | Bt03210471_g1 | Bt.30099 |
| FOXO3 | Forkhead Box O3 | Bt03649334_s1 | Bt.44365 |
| GAPDH | glyceraldehyde-3-phosphate dehydrogenase | Bt03210912_g1 | Bt.87389 |
| GPX1 | Glutathione Peroxidase 1 | Bt03259217_g1 | BT.4317 |
| HSPA1A | Heat shock 70kDa protein 1A | Bt03292670_g1 | Bt.49659 |
| NANOG | Nanog homeobox | Bt03220541_m1 | BT.47449 |
| NFE2L2 | Nuclear factor (erythroid-derived 2)-like 2 | Bt03251880_m1 | BT.17324 |
| NOS2 | Nitric oxide synthase 2 | Bt03249586_m1 | Bt.23126 |
| PA2G4 | Proliferation-Associated 2G4 | Bt03211241_g1 | Bt.106792 |
| POU5F1 | POU class 5 homeobox 1 | Bt03223846_g1 | BT.92603 |
| PPARG | Peroxisome Proliferator Activated Receptor Gamma | Bt03217547_m1 | Bt.3862 |
| PPARα | Peroxisome Proliferator Activated Receptor Alpha | Mm00627559_m1 | Mm.212789 |
| PPIA | Peptidylprolyl Isomerase A | Bt03224617_g1 | Bt.43626 |
| PTGS2 | Prostaglandin-endoperoxide synthase 2 | Bt03214492_m1 | Bt.15758 |
| SALL4 | Spalt-Like Transcription Factor 4 | Bt04298191_g1 | Bt.8493 |
| SCD | Stearoyl-CoA desaturase | Bt04307477_m1 | Bt.65021 |
| SOX2 | SRY (sex determining region Y)-box 2 | Bt03278318_s1 | Bt.103364 |
| TXNRD1 | Thioredoxin Reductase 1 | Bt03215471_m1 | Bt.5534 |
