# Supplementary material for: Genome-wide screening of DNA methylation in bovine blastocysts with different kinetics of development
Source: Epigenetics Chromatin. 2018 Jan 8;11:1. doi: 10.1186/s13072-017-0171-z (PMC5757301; doi:10.1186/s13072-017-0171-z)
Supplement: Supplementary file 3 — Additional file 3. Hypermethylations in FBL. [file 13072_2017_171_MOESM3_ESM.docx]

| **Biological Process Hypermethylated in FBL** | | | |
| --- | --- | --- | --- |
| Term | Count | PValue | Genes |
| Phosphate metabolic process | 134 | 2.21E-09 | STK38, EFNA1, DSTYK, PKMYT1, PTPN21, LPAR1, PRKG1, TGFB2, CLK3, AAK1, ILK, PRKACB, INSR, PTPRF, ROCK1, PTPRG, PTPRN2, MYLK4, MYO3B, PTPRR, PTPRT, PTPRU, STK3, MAPK6, CAMK1, MAPK9, ROR2, TESK2, CSNK1G3, EIF2AK2, LRRK1, NEK6, KALRN, FGFR1, SSH1, STK10, SSH2, MAPKAPK3, ADRBK2, ADRBK1, DUSP12, EPHA10, MAPKAPK2, ATP6V1B2, STK17A, PXK, MST4, EPHB1, CAMKV, DUSP14, DUSP15, ITK, TGFBR1, MAP2K4, OXSR1, GUCY2C, PPA2, DYRK1B, SPTBN1, GRK5, PTPN1, MERTK, MYLK, IMPA1, UQCRC1, NUAK2, EIF2A, DDR2, ACVR1B, PDPK1, PKN3, PAK4, SYNJ2, PAK1, CDK16, ATP6V0D1, SIK2, SIK3, CHUK, IRAK2, PRKCA, TRPM6, SGK1, ADAM10, SGK2, LYN, CDK8, PI4KA, PRKCI, PRKCH, PKN1, DAPK2, PRKCE, DAPK3, NEK11, TBCK, GAK, MAST3, HUNK, PDIK1L, DDR1, PPM1G, KSR2, HIPK3, RIPK1, HIPK2, TXK, PPM1L, BRSK2, MKNK1, CSNK1B, PTPMT1, KIT, BRSK1, TRIB2, MTMR2, GALK2, STK32A, STK40, BCL2, CAMK2D, DCLK2, PPP3CA, LOC614815, YES1, MTMR4, PDK2, FLT1, MAPK10, TEX14, FYN, CSNK1E, ATP5A1, CIT |
| Intracellular signaling cascade | 83 | 0.006161 | FGF8, PLEKHM3, STK38, EFNA1, WASF2, IQGAP3, LPAR1, RBM9, DAXX, STAC, RAB28, NMUR2, RALB, RAB24, RHOC, RAB6A, AGAP1, PLCB1, RAB27B, INSR, SIK2, NET1, PRKCA, ARHGEF3, PTGER3, LYN, ROCK1, ARHGEF5, PI4KA, PRKCH, RASL12, PRKCE, DAPK3, DCDC1, ELMO1, ARHGEF11, KSR2, SGSM3, UBR5, ASB1, RAB12, PPM1L, TXK, TSHR, LRRK1, SPATA13, UNC13A, CALCR, RAB7B, PLEK2, MRAS, MKNK1, AKAP13, KIT, ABCA1, LOC616969, PLCL2, LOC515718, PLCH2, PLCH1, APOC3, PKD2, DCLK2, INPP5D, ITK, RAB8A, RABIF, TGFBR1, RCAN1, OXSR1, MAPK10, GUCY2C, TAX1BP3, RCAN2, RABL2B, RASL11B, RERG, MED30, PLCG1, RAB35, RGS6, BRE, CIT |
| Ion transport | 80 | 0.030787 | SLC9A9, KCNC2, SLC8A3, KCNH1, GRIK1, SLC20A2, GABRB2, GRIK3, GABRB1, LOC531152, KCNJ10, GRIN3B, CNGB3, KCNQ4, KCNQ3, GRIN2B, NMUR2, VPS4B, SCN7A, ANO4, ATP6V0D1, SLC22A1, KCNMA1, GABRG3, TRPM6, KCND2, PTGER3, CFTR, SLC34A2, CATSPER4, ATP2C2, NNT, GABRR1, RHCG, CLIC4, CLIC5, RYR3, KCNH2, CLDN16, KCNA2, CACNB2, ATP6V1G2, CACNB3, ATP6V1B2, ATP12A, TPCN1, TPCN2, ANXA6, CAMK2D, PKD2, PPP3CA, SLC31A2, SLC31A1, NFATC1, GABRE, GRIA3, SLC10A4, GRIA4, LOC783993, KCTD5, ITPR3, KCNK1, SLC9A10, KCNK2, ATP13A5, ITPR1, KCNV1, SLC10A1, KCNJ5, CYBB, KCNJ6, SCN4B, SLC13A3, CACNA1E, SLC5A7, ATP5A1, LOC526524, CLCN7, CACNA1D, CACNA1B |
| Macromolecule catabolic process | 55 | 0.022168 | CUL3, CUL2, PARN, RBM8A, WWP2, WWP1, PGLYRP3, NSMCE2, RANBP2, USP13, CLN3, ADAM10, FBXL21, DFFB, UBE2H, UBR1, MYH9, UBE2N, UBR5, RNF25, FBXL4, USP20, YME1L1, FBXO15, USP25, UBE2U, RAD23B, DERL1, LOC539472, USP4, PPT1, PSMB11, PSMB5, CACYBP, RNF128, FBXO6, USP38, HECTD3, RNF168, UBE2D1, USP34, ERCC3, USP33, TRIP12, USP32, RNF144B, OVGP1, UBE2CBP, AFG3L2, MANBA, LOC529686, NCSTN, VCP, UBA1, UBA2 |
| Regulation of programmed cell death | 51 | 0.020638 | FGF8, MAEA, PDIA3, RBM5, CTNNBL1, TGFB2, GPX1, BDNF, NLRC4, TICAM1, TMEM102, DDX20, NDUFS3, PRKCA, KCNMA1, CLN3, AIFM2, HRK, RAD9A, CSDA, BCL2L13, DAPK3, CARD10, ALOX15, RIPK1, IL12A, MAPK9, NGFR, IL12B, PPT1, ZBTB16, BDKRB2, KIT, ADA, BCL2, FCGR1A, LHX4, FAIM, INPP5D, ERCC3, RASA1, COL4A3, IL7, TMBIM6, TGFBR1, EEF1A2, MAPK10, SON, PSMG2, VCP, CFDP1 |
| Regulation of cell proliferation | 47 | 0.026898 | FGF18, FGF8, PPARD, PDGFB, PTGS2, PDGFA, IL18, PTGS1, PPARG, KIT, ZBTB16, CXADR, DDR2, ADA, CD9, GPX1, BDNF, GPC3, BCL2, ILK, TICAM1, INPP5D, FGF1, FIGF, INSR, PRKCA, ADAM10, IL7, TGFBR1, RPS9, ESR2, TAX1BP3, GUCY2C, FOXP1, RERG, DDR1, HHEX, ALOX15, PLA2G4A, IL12A, CFDP1, PTCH1, NGFR, ATP5A1, IL12B, SMARCA2, PLAU |
| **Cellular componentes Hypermethylated in FBL** | | | |
| Plasma membrane | 171 | 0.002299 | F2RL2, KCNC2, MICB, MAEA, OCLN, GRIK1, SLC6A1, EFNA1, GABRB2, SLC6A2, GABRB1, SLC6A4, PTGS1, LPAR1, AMOTL2, MMP2, ADORA1, GHRHR, SYP, LPHN2, MALL, SLC2A4, RAB28, ILK, SLC2A1, PRKACB, CDH26, INSR, CDH23, F11R, KCND2, LOC616741, PICK1, ACTN2, BASP1, MYH9, TRAT1, SLC34A2, NPC1, LYNX1, BSC1, RHCG, SGSM3, CCR3, TMPRSS11F, ROR2, VAMP1, GRB14, ITGA2B, BBS4, ARFGAP2, KCNA2, CACNB2, ITGB5, SNX1, CACNB3, EPHA10, ABCA1, ATP6V1B2, PXK, NRN1, ADA, EPHB1, PPP1R16B, LOC515718, PCDHA13, AP2M1, RAB8A, SLC6A16, SLC6A17, UPK3A, HOMER1, KCTD5, KCNK2, CAMK2N1, LIN7A, ITGA9, LAMP1, KCNJ6, CDC42SE2, ITGA5, RGS6, S100G, SPTBN1, CACNA1E, GRK5, CACNA1D, ARHGAP10, MYH10, CACNA1B, MCHR1, SLC27A1, CLDN7, RGS7BP, GPR161, SLC20A2, CRCP, CXADR, MBP, EDNRA, WISP2, KCNQ4, AP2B1, KCNQ3, NMUR2, CXCR6, SMAGP, NECAP1, RHOC, SCN7A, CAP1, SLC22A1, GHR, PRKCA, KCNMA1, ICAM1, CLN3, ADAM10, PTGER3, PTGER4, PRKCI, LRPAP1, ELMO1, DDR1, FOLH1, GRM3, PFDN1, CHRM3, CLIC5, RIPK1, NK1R, NGFR, THEM4, C15H11ORF59, TSHR, SNAP29, CLDN16, CALCR, CPLX3, CD247, SYNC, ABI2, CDH2, KIT, CDH3, CDH4, DTNBP1, FAT3, SORBS1, FCGR1A, CAMK2D, PKD2, SNAP25, RASA1, MARS, FLT1, SWAP70, GRIA4, ITPR3, KCNV1, NCSTN, COG4, CYBB, FYN, LRP6, SLC5A7, ATP5A1, YKT6, LRP4, NTM, SNTA1 |
| Organelle lumen | 88 | 0.00956 | FGF18, KDM6A, MAEA, PDIA3, STK38, LOC535415, LMO4, PEX5, PDIA5, OGDH, DAXX, WDR74, INTS9, FOS, RBM8A, DDX20, TBPL1, PDPR, SATB1, MRPL51, EXOSC8, MRPL3, POLR1E, YY1, BYSL, OTC, APTX, EXOSC3, ACTN2, EXOSC1, ECSIT, SARS2, RDH5, TUT1, HAGH, RCL1, COQ3, C1QBP, ALDH1B1, SIX1, SIL1, TSG118, CLN5, BAT1, SUPT3H, LOC517559, WARS2, ZBTB16, PIN4, HADHA, SDHAF1, CALU, MINA, MRPL11, MRPL10, MRPL13, MRPL16, BRIX1, RSL24D1, ETV6, ERCC3, GEMIN7, NSA2, BCKDHA, POLR3H, EPAS1, PNO1, SRA1, BCKDHB, MAML2, RPS9, STAT1, ITPR3, ITPR1, MED31, PHF17, MGC134577, PPIH, PKNOX1, PCNA, RBM19, SPTBN1, POP4, NOP56, RCN3, PARP1, RBPJ, RPP40 |
| Endoplasmic reticulum | 65 | 0.04477 | IER3IP1, HM13, PDIA3, PTGS2, LMAN2L, PTGS1, PDIA5, DSE, CISD2, PTGIS, GLT25D2, PIGF, RESP18, POMT1, INSIG1, SCD5, ELOVL6, DDOST, CLN3, SGK1, SPTLC1, ACO1, PIGV, DPAGT1, TMEM189, SCAP, LRPAP1, RDH5, TOR2A, NPC1, DGAT1, DGAT2, SIL1, HAUS8, DOLPP1, GULO, EXT1, CLN5, EXTL3, DERL1, EXTL1, CALU, BCL2, RNF128, PPAP2B, HSD17B7, NSDHL, SOAT1, GABARAPL1, HERPUD1, CAMLG, FADS3, UPK3A, ITPR3, LOC777692, ITPR1, NCSTN, TXNDC11, VCP, LASS4, LASS2, RCN3, LOC526524, YKT6, SSR2 |
| Golgi apparatus | 61 | 0.006391 | SH3RF1, IER3IP1, KDM6A, NSG2, LMAN2L, PITPNB, PTGS1, PRKG1, SLC35A1, CXADR, DSE, AP2B1, MALL, ST3GAL2, SLC2A4, ST3GAL5, GALNTL4, RESP18, RAB6A, RAB27B, SEC24D, STX6, CLN3, STX5, ADAM10, GOLT1A, ROCK1, BEND5, ACO1, PTPRR, SCAP, MGAT1, CHPF, CLIC5, CHSY1, EXT1, CLN5, GRB14, ARFGAP2, MFNG, GALNT6, SNX1, PPT1, ABCA1, ATP6V1B2, LMAN1, RRAGB, B3GNT5, RNF128, TMED10, NDRG2, SNAP25, GABARAPL1, LAPTM4A, SOD3, NCSTN, COG4, KCNJ6, COG6, ST8SIA5, YKT6 |
|  |  |  | **Mollecular Functions Hypermethylated in FBL** |
| Ion binding | 388 | 7.47E-06 | FHIT, LTBP2, MASP1, PTGS2, PTGS1, ZNF250, RORA, SYP, BRPF1, ZNF394, CDH26, CDH23, ZNF641, CRTAC1, TADA2B, ZHX1, ZNF48, RXRG, COLEC12, SSPO, OGFOD1, LOC536660, ZNF235, RYR3, ADAMTS1, ADAMTS2, MST1, CACNB2, CACNB3, DIDO1, LPCAT1, ARG2, ZC3H14, NPLOC4, ESRRB, TGFBR1, ESRRG, ATP11A, ATP11C, KCNK1, MARCH10, MARCH11, LOC511936, LOC540707, GAS6, ZNF629, ZNF526, CYP7B1, ADAP2, LOC100125388, TRAFD1, PARP1, TLL2, ZNF385A, RERE, KCNH1, SH3RF1, CYP2J2, SLC20A2, ZNF296, TP63, RNF187, NUBP1, MGRN1, SIK2, GHR, USP13, ZDHHC3, ZC3H7A, ACO1, MICAL2, SLC3A2, CFTR, ZNF143, SLC3A1, ZNF689, ZFR2, ZSWIM3, MAST3, ALOX15, MYRIP, TAF15, CLIC4, CLIC5, ZNF277, USP20, AICDA, RNPEP, ZNF274, REPS1, TRIM14, NOB1, ZNF35, ZNF75A, PCSK1, TCEA3, MMACHC, PITRM1, PKD2, PPP3CA, TCEA2, USP33, USP32, ZNF263, ZNF454, ZNF771, LOC519314, FADS3, ZFP1, ZNF667, PHF10, ZNF25, PDZRN3, MARCH7, MARCH6, MANBA, PHF17, CDH15, ZNF672, CDH17, BNC2, BNC1, SH3RF2, LRP8, LOC615051, LRP4, LMO1, GMPR2, STK38, LMO3, LMO4, RBM5, RPS27L, MOBKL2C, MOBKL2B, APOA4, ZNF446, RNF145, MTA3, ACTN2, CDO1, SLC34A2, RNF130, KDM2A, MIB2, VSNL1, ZNF438, XDH, ARFGAP2, PAM, ME3, ME2, KCNA2, NT5C1B, LOC534495, CRB1, RNF168, ZNF420, PCDHA13, FGD6, ADAM28, OVGP1, CUBN, TRIP4, EHMT1, WHSC1, P4HTM, MAN2C1, ATF7, S100G, CACNA1E, SPTA1, CACNA1D, CACNA1B, IMPA1, APLF, HEXA, TRIM2, ANO4, LOXL2, SEC24D, ZCCHC7, RTN4IP1, MBNL1, ESR2, HAGH, PPM1G, RFWD3, PKM2, CA6, CA3, PPM1L, CPSF3, DPF2, DPF3, ADAMTS17, GALNT6, ABLIM3, ADAMTS16, CDH2, LOC616969, CDH3, CDH4, ANXA6, RNF126, ANXA9, PRRG1, NUDT7, RNF128, CHD5, RNF144B, CREB5, ANXA5, ISL1, ANXA4, CYBB, ZNF397OS, KDM4C, SCN4B, ZRANB3, RCN3, SNTA1, QPCTL, ZNRF1, STAC, SCD5, RNF220, YY1, UBR1, LOC786435, PIAS3, PGM1, ADD1, FUS, LOC531175, ENPP2, FARS2, ASAP1, ASAP3, RIMS1, ADA, CALU, PLAGL1, DMD, LHX4, AGBL5, CREBBP, RNF207, OXSR1, AFG3L2, PPA2, CYP4B1, ZFHX4, PLA2G4A, RNF4, LOC787809, CLCN7, PPARA, PPARD, ZMAT4, NPNT, PPARG, KCNJ10, GLI2, WBP4, PARN, NSMCE2, AGAP1, KCNMA1, ADAM10, SF1, RPH3AL, APTX, RAD50, RAB11FIP4, ADAM12, MECR, ADAM15, CLDN16, CAPS, CETN2, CETN1, ZBTB16, EDEM2, PLCL2, FAT3, SQSTM1, ETFDH, CHP, THBS2, THBS4, BCKDHA, TBXAS1, VWCE, ITPR3, STAT1, ITPR1, KCNV1, SOD3, GBA3, YAF2, FYN, FBLN5, PHF21B, FBLN7, MEX3C, CALM3, NLN, VLDLR, GABRB2, GABRB1, F13A1, SFTPA1B, MMP2, ZNF184, ZNF181, CISD2, PTGIS, SLC25A25, DNTT, TRIM45, LOX, MYST3, TRIM37, TRIM33, RNF25, ANKFY1, LOC789528, ZC3H3, EFCAB6, AGFG2, ZMYND8, PLCH2, RNFT2, PLCH1, EFCAB1, HEG1, ENTPD1, CYP19A1, SETDB2, RABIF, MYLPF, LOC783993, LOC789845, FOXP1, MSL2, DYTN, LEPRE1, PLCG1, PRICKLE1, PRICKLE2, MYLK, UQCRC1, ZNF81, NR6A1, PHC3, PCGF5, KCNQ3, GALNTL4, ATP8B2, RANBP2, PLCB1, ATP8B4, PRKCA, LOC617365, C13H20ORF12, EGR4, PRKCH, TUT1, MDM2, MDM4, LEPREL1, MATR3, ADPGK, SPOCK1, CPZ, MYL6B, LOC521092, SFRS2IP, LOC522441, ZNF605, ZC3H12C, MAP1D, DTX2, MGC159954, SP2, ATXN7, LTA4H |
| Nucleotide binding | 273 | 3.13E-06 | RBPMS2, PGD, SART3, PRKG1, CLK3, RAB28, SFRS9, ILK, VPS4B, RAB24, VPS4A, RAB27B, DHX32, ROCK1, LIG1, SUCLG1, KIF5C, MYLK4, OLA1, MYH9, SARS2, BOLL, LOC536660, ATP2C2, NNT, MAPK6, LOC518080, RFC2, SMARCAL1, ROR2, MAPK9, CAMK1, YME1L1, RAB12, EIF2AK2, EEFSEC, NEK6, RALYL, FARS2, TDRD9, PFKFB1, ADRBK2, WARS2, IGF2BP2, ADRBK1, STK17A, PXK, NAGK, ATP12A, MST4, EPHB1, CAMKV, DHX57, DCAKD, KIF3B, KIF3A, TGFBR1, MAP2K4, ATP11A, ACACB, ATP11C, OXSR1, AFG3L2, GUCY2C, DDX4, ATP13A5, CCT7, RERG, UBA1, UBA2, CCT8, RAD54B, GRK5, PARP1, CLCN7, SLC27A1, ABCF2, NUAK2, CPEB4, CRCP, ANKRD17, PDPK1, PARN, NUBP1, NMUR2, SYNJ2, RAB6A, AGAP1, SIK2, SIK3, AIFM2, LYN, PI4KA, CFTR, RAD50, RAD51, HNRPDL, MAST3, PDIK1L, KSR2, TAF15, RIPK1, MGC154956, TXK, ENOX1, CSNK1B, MKNK1, CETN2, CETN1, KIT, GALK2, STK40, DDX19B, HK3, CKMT1, DHX15, ACTL6B, ACTL6A, ABCA13, RBM25, MARS, PDK2, LOC781070, SWAP70, MAPK10, ELAVL3, PCK2, ELAVL4, SDHA, TEX14, CSNK1E, FYN, RAB35, RBM19, ATP5A1, CIT, GNA14, MYH15, STK38, DTYMK, RBM5, PKMYT1, DSTYK, FOX1, RBM9, IGHMBP2, AAK1, RALB, PRKACB, DDX20, INSR, MTO1, LOC614209, MYO3B, STK3, RBPMS, EFTUD1, DDX31, TESK2, CSNK1G3, GULO, SMARCA2, LRRK1, RAD17, PCCA, KALRN, BAT1, XDH, FGFR1, RAB7B, ME3, ME2, LOC514078, BLM, STK10, MRAS, NT5C1B, MAPKAPK3, LOC524159, EPHA10, ABCA1, MAPKAPK2, RRAGB, RAD51L1, ENTPD1, UBE2D1, ITK, RAB8A, MYO1B, EEF1A2, MYO1E, DOCK9, MYO1G, RIMKLA, PPIE, MGC134577, MYO10, VCP, DYRK1B, MYO16, MYH14, FPGS, DDX54, MERTK, MYLK, MYH10, APLF, DDR2, ACVR1B, PKN3, PAK4, ATP8B2, RHOC, PAK1, CDK16, CHUK, ATP8B4, IRAK2, PRKCA, KIF14, SGK1, TRPM6, SGK2, KIF17, NOL8, PRKCI, CDK8, G3BP2, PKN1, PRKCH, UBE2H, KIF16B, PRKCE, DAPK2, RASL12, DAPK3, TBCK, NEK11, GAK, TUT1, UBE2N, TOR2A, DDR1, HUNK, HIPK3, HIPK2, TOM1, MATR3, FKBP4, TRA2A, BRSK2, BRSK1, TRIB2, STK32A, TNRC6C, SYN3, SYN2, CAMK2D, LARS, LOC512287, DCLK2, LOC614815, TNRC6B, YES1, ERCC3, CHD5, GNAO1, FLT1, GIMAP5, RABL2B, RASL11B, CYBB, ZRANB3 |
| ATP binding | 199 | 2.58E-09 | PRKG1, CLK3, ILK, VPS4B, VPS4A, DHX32, ROCK1, LIG1, KIF5C, MYLK4, OLA1, MYH9, SARS2, LOC536660, ATP2C2, LOC518080, MAPK6, RFC2, SMARCAL1, MAPK9, CAMK1, ROR2, YME1L1, EIF2AK2, NEK6, FARS2, TDRD9, PFKFB1, ADRBK2, ADRBK1, WARS2, STK17A, PXK, ATP12A, NAGK, MST4, EPHB1, CAMKV, DCAKD, DHX57, KIF3B, KIF3A, TGFBR1, MAP2K4, ATP11A, ATP11C, OXSR1, ACACB, GUCY2C, AFG3L2, DDX4, ATP13A5, CCT7, UBA1, UBA2, CCT8, RAD54B, GRK5, CLCN7, ABCF2, NUAK2, ANKRD17, PDPK1, NUBP1, SIK2, SIK3, LYN, PI4KA, CFTR, RAD50, RAD51, MAST3, PDIK1L, KSR2, RIPK1, TXK, MKNK1, CETN2, CSNK1B, CETN1, KIT, GALK2, STK40, HK3, DDX19B, CKMT1, DHX15, ACTL6B, ACTL6A, ABCA13, MARS, PDK2, SWAP70, MAPK10, TEX14, FYN, CSNK1E, ATP5A1, CIT, MYH15, STK38, DTYMK, DSTYK, PKMYT1, IGHMBP2, AAK1, DDX20, PRKACB, INSR, MYO3B, STK3, TESK2, DDX31, CSNK1G3, SMARCA2, LRRK1, RAD17, PCCA, KALRN, BAT1, FGFR1, LOC514078, BLM, STK10, MAPKAPK3, LOC524159, EPHA10, ABCA1, MAPKAPK2, RAD51L1, UBE2D1, ENTPD1, ITK, MYO1B, MYO1E, MYO1G, RIMKLA, MYO10, VCP, DYRK1B, MYO16, MYH14, DDX54, FPGS, MERTK, MYLK, MYH10, DDR2, ACVR1B, PKN3, PAK4, ATP8B2, PAK1, CDK16, CHUK, ATP8B4, IRAK2, KIF14, PRKCA, TRPM6, SGK1, SGK2, KIF17, PRKCI, CDK8, PKN1, PRKCH, KIF16B, UBE2H, DAPK2, PRKCE, DAPK3, TBCK, NEK11, GAK, TOR2A, UBE2N, HUNK, DDR1, HIPK3, HIPK2, TOM1, FKBP4, BRSK2, BRSK1, TRIB2, STK32A, SYN3, SYN2, LARS, CAMK2D, LOC512287, DCLK2, LOC614815, YES1, ERCC3, CHD5, FLT1, ZRANB3 |
