# Supplementary material for: Genome-wide screening of DNA methylation in bovine blastocysts with different kinetics of development
Source: Epigenetics Chromatin. 2018 Jan 8;11:1. doi: 10.1186/s13072-017-0171-z (PMC5757301; doi:10.1186/s13072-017-0171-z)
Supplement: Supplementary file 4 — Additional file 4. Hypermethylations in SBL [file 13072_2017_171_MOESM4_ESM.docx]

| **Biological Process Hypermethylated in SBL** | | | |
| --- | --- | --- | --- |
| Term | Count | PValue | Genes |
| Phosphate metabolic process | 55 | 0.026609 | BCKDK, NRBP1, PRPF4B, PINK1, FER, ADCK1, MAP3K4, PRKACA, ATP5O, LOC787471, ATP6V0D2, MAP2K6, EGFR, SRPK2, SGK3, CDK4, MARK4, STK4, PRKCB, CDKL4, MARK2, ACVR2A, EYA2, HIPK4, TNNI3K, RAGE, NEK4, MAP3K12, FGFR2, STYXL1, NEK3, MVD, MAPKAPK5, DUSP10, RIOK1, ATP5G3, EPHB2, GPD1L, DUSP18, STK32C, CDC2L1, RYK, MAP2K2, MAK, EPM2A, MET, TRIO, RPS6KL1, GLYCTK, RPS6KA5, LOC616014, PLK1, RPS6KA2, EPHA8, ATP6V1E1, ABL2 |
| RNA processing | 27 | 0.020994 | PRPF4B, ZMAT5, STRAP, U2AF2, UTP15, MLH1, BOP1, PNN, ADAT1, EXOSC10, SBDS, MOV10, CTU1, HNRNPF, U2AF1, RPL11, SNRNP70, TSEN2, LOC518878, RBM23, RRP1, FBL, JMJD6, SNRPB, POP5, PRPF38A, ADAR |
| Regulation of small GTPase mediated signal transduction | 24 | 2.89E-04 | LOC521706, ABR, AGFG1, RAP1GAP, ARHGEF7, PREX1, ARHGEF19, ASAP2, TRIO, ARFGEF1, FARP1, TBC1D22A, MCF2L, PLEKHG1, TSC1, TBC1D14, RAPGEF5, RAPGEF3, RASA3, TBC1D30, RAPGEF2, RASA4, ARHGEF10L, IQSEC2 |
| Generation of precursor metabolites and energy | 20 | 0.085287 | ALDOA, DLST, ACOX1, PFKL, NDUFA8, COX10, OGDHL, NDUFA10, ATP5G3, LOC613316, GCK, ATP6V1E1, NDUFV1, GAA, ENO3, ATP5O, SUCLA2, ATP6V0D2, MDH2, PYGB |
| Regulation of Ras protein signal transduction | 18 | 0.002816 | LOC521706, ABR, AGFG1, ARHGEF7, PREX1, ARHGEF19, ASAP2, TRIO, ARFGEF1, FARP1, TBC1D22A, MCF2L, PLEKHG1, TSC1, TBC1D14, TBC1D30, ARHGEF10L, IQSEC2 |
| Chordate embryonic development | 17 | 0.05676 | FGFR2, BMP4, XRCC4, PRRX1, ACVR2A, SBDS, PSMC4, TSC1, NCAPG2, CDC2L1, HOXD4, GATAD2A, DAD1, HOPX, NLE1, SMARCA4, MGC142792 |
| Cellular component morphogenesis | 15 | 0.031323 | EGFR, SHROOM1, EZR, AGFG1, NTF3, COX10, PDPN, CXCR4, MYBPC3, RHOA, ETV1, APBB2, STK4, CACNA1A, MGC142792 |
| Secretion | 14 | 0.04175 | PLDN, CAV1.2, ADORA3, GLRA1, LIN7B, STXBP3, AGXT, FKBP1B, SCFD2, STEAP2, UNC13B, CHRNA3, CACNA1A, SCAMP5 |
| **Cellular componentes Hypermethylated in SBL** | | | |
| Plasma membrane | 88 | 0.015714 | KCNC1, TLN1, ADORA3, GLRA1, VAPB, F2RL1, CLDN5, TLR2, UNC93A, VIPR1, SPRY4, SCTR, PNN, HMOX2, COPB2, DYSF, MC1R, CXCR4, LTB4R, RHOA, ITIH4, MS4A1, ATP5O, PRKACA, KCNG4, ATP6V0D2, CHRNA3, LPHN1, SCAMP5, EGFR, CAPNS1, CAV1.2, CCKBR, GPR18, ZP3, PDPN, PCDH11X, CDHR2, STIM1, CTNNA2, ACVR2A, COPG2, FAM120A, ST14, LOC616071, STEAP2, EDA, SEZ6, MGC142792, GPR183, DRD1, CALY, OSTA, ALPI, CRIPT, ADCYAP1R1, NPY2R, LY6G6C, CALR, EPHB2, EZR, UPK1B, ITGB6, ADRA2A, EHD2, RAB8B, SLC12A3, KCNB2, RYK, LMNA, LIN7B, ATP1A1, ANKH, CAPN1, CDH13, VWF, AP2A2, TMEM8B, EPHA8, JMJD6, GNG10, ATP6V1E1, DSG1, SLC6A6, ABCC1, KCTD13, CACNA1A, MTNR1A, DNM2 |
| Mitochondrion | 58 | 0.017402 | ACOX1, BCKDK, D2HGDH, TUSC3, SNCB, COX10, HINT2, BNIP3, MTHFD1, ACOT8, KBP, HTRA2, MTG1, MP68, ATP5O, PRKACA, ACAD8, SUCLA2, OMA1, OPA3, PISD, NDUFA10, LOC613316, COQ2, SLC25A34, MTRF1, RDBP, TXNRD2, PMPCA, MDH2, MRPS16, ECHS1, CHCHD4, MTIF2, ATP5G3, DCI, HADHB, MRPL19, SLC25A45, NTHL1, GAD1, ACSL5, DLST, NDUFA8, MRPS22, AMACR, TOMM40, CRAT, SLC25A13, PSMC4, TMEM8B, NDUFV1, ATP6V1E1, GOLPH3, FABP3, HIBCH, SPNS1, LRP5 |
| Endoplasmic reticulum | 34 | 0.095871 | DRD1, TUSC3, VAPB, MRVI1, RCE1, CALR, FKBP1B, CANX, OS9, MOV10, AQP11, PEX16, LRRTM1, UPK1B, VKORC1, DAD1, PCYT1A, TSEN2, PLD3, CTSZ, MOGAT1, VAC14, SCD, STIM1, LPCAT3, PNPLA6, VWF, EBPL, TRAPPC9, TMEM8B, SDF2L1, C3ORF57, EDA, FKBP2 |
| Golgi apparatus part | 14 | 0.041585 | SCAMP3, ST6GALNAC1, ST6GALNAC5, CSGALNACT1, ST3GAL1, COPB2, COPG2, ST3GAL3, COG7, CHST7, ST3GAL4, GOLPH3, STEAP2, SCAMP5 |
|  |  |  | **Mollecular Functions Hypermethylated in SBL** |
| Nucleotide binding | 135 | 9.46E-04 | RALY, PRPF4B, NRBP1, U2AF2, MAP3K4, ATP2B4, MTG1, RAVER1, LOC540047, U2AF1, PRKACA, ITPK1, MAP2K6, KIF13B, EGFR, YARS, GTPBP5, CSTF2T, STK4, MARK4, MARK2, CDKL4, NAV2, RDBP, NEK4, EDA, PRPS2, EP400, SMARCA4, FGFR2, DNAH11, MVD, GNAI2, NEK3, ACTR3B, MAPKAPK5, RAB40C, CTPS2, IGF2BP3, RIOK1, MTIF2, KARS, CDC37, ABCA6, EPHB2, GPD1L, MOV10, HNRNPF, SFRS11, LOC518878, LOC616722, RAB8B, RYK, MAP2K2, MYO1D, MET, MYO1F, TRIO, DOCK6, RPS6KL1, GLYCTK, UBE2E3, DHFR, PSMC4, PLK1, ATP2A3, EPHA8, POLD1, NDUFV1, GTF2F2, MYH11, MYO19, ABL2, CRYM, ATP7B, ACOX1, BCKDK, D2HGDH, SEPHS1, CPEB3, PINK1, MLH1, FER, MTHFD1, ADCK1, CTU1, RHOA, LOC528767, ACAD8, SUCLA2, TUBA1B, TUBB3, SRPK2, AIFM3, PFKL, SGK3, NDUFA10, CDK4, PRKCB, GNAL, ACVR2A, TNNI3K, HIPK4, TXNRD2, RUVBL2, TXNRD1, KIF19, RAGE, STEAP2, MAP3K12, POLR2D, DNAH7, STK32C, PARS2, CDC2L1, MSI2, TUBA3E, SNRNP70, EHD2, PAPSS2, RBM23, MAK, GIMAP8, ATP1A3, ATP1A1, SIRT1, RPS6KA5, LOC616014, GSPT1, GCK, RPS6KA2, AOX1, RAB34, ABCC1, DPYD, DNM2 |
| ATP binding | 84 | 0.015331 | BCKDK, PRPF4B, SEPHS1, NRBP1, PINK1, MLH1, FER, MTHFD1, ADCK1, ATP2B4, MAP3K4, CTU1, LOC528767, PRKACA, SUCLA2, ITPK1, MAP2K6, KIF13B, EGFR, SRPK2, YARS, SGK3, PFKL, NDUFA10, CDK4, STK4, MARK4, PRKCB, CDKL4, MARK2, ACVR2A, TNNI3K, HIPK4, KIF19, RUVBL2, RAGE, NEK4, MAP3K12, PRPS2, EP400, SMARCA4, DNAH11, FGFR2, NEK3, MVD, ACTR3B, MAPKAPK5, CTPS2, RIOK1, DNAH7, ABCA6, KARS, CDC37, EPHB2, STK32C, MOV10, PARS2, CDC2L1, PAPSS2, MAK, RYK, MAP2K2, MYO1D, MET, ATP1A3, MYO1F, TRIO, ATP1A1, RPS6KL1, GLYCTK, RPS6KA5, LOC616014, UBE2E3, GCK, PSMC4, PLK1, ATP2A3, EPHA8, RPS6KA2, GTF2F2, MYH11, ABCC1, MYO19, ABL2, ATP7B |
| Protein kinase activity | 44 | 0.002111 | FGFR2, BCKDK, NRBP1, PRPF4B, NEK3, MAPKAPK5, PINK1, FER, RIOK1, EPHB2, STK32C, ADCK1, MAP3K4, CDC2L1, PRKACA, MAP2K6, EGFR, SRPK2, SGK3, MAP2K2, RYK, MAK, MET, TRIO, ATR, CDK4, MARK4, STK4, RPS6KL1, PRKCB, MARK2, CDKL4, RPS6KA5, LOC616014, ACVR2A, CCND3, RPS6KA2, PLK1, EPHA8, HIPK4, TNNI3K, RAGE, NEK4, ABL2, MAP3K12 |
